# Clinical and biochemical characteristics of people experiencing post-coronavirus disease 2019-related symptoms: A prospective follow-up investigation

**DOI:** 10.3389/fmed.2022.1067082

**Published:** 2022-12-06

**Authors:** Assim A. Alfadda, Mohamed Rafiullah, Mohammad Alkhowaiter, Naif Alotaibi, Musa Alzahrani, Khalifa Binkhamis, Khalid Siddiqui, Amira Youssef, Haifa Altalhi, Ibrahim Almaghlouth, Mohammed Alarifi, Saleh Albanyan, Mohammed F. Alosaimi, Arthur Isnani, Shaik Sarfaraz Nawaz, Khalid Alayed

**Affiliations:** ^1^Strategic Center for Diabetes Research, College of Medicine, King Saud University, Riyadh, Saudi Arabia; ^2^Obesity Research Center, College of Medicine, King Saud University, Riyadh, Saudi Arabia; ^3^Department of Internal Medicine, College of Medicine, King Khalid University Hospital, King Saud University Medical City, King Saud University, Riyadh, Saudi Arabia; ^4^Department of Medicine, College of Medicine, King Saud University Medical City, King Saud University, Riyadh, Saudi Arabia; ^5^Department of Medicine, College of Medicine, King Saud University, Riyadh, Saudi Arabia; ^6^Department of Pathology, College of Medicine, King Saud University, Riyadh, Saudi Arabia; ^7^Infection Control Department, King Khalid University Hospital, King Saud University Medical City, Riyadh, Saudi Arabia; ^8^Rheumatology Department, King Khalid University Hospital, King Saud University Medical City, Riyadh, Saudi Arabia; ^9^Intensive Care Department, King Khalid University Hospital, King Saud University Medical City, Riyadh, Saudi Arabia; ^10^Department of Pediatrics, King Khalid University Hospital, King Saud University Medical City, Riyadh, Saudi Arabia

**Keywords:** SARS-CoV-2, COVID-19, long COVID, post-COVID-19, PASC

## Abstract

**Background:**

Post-acute coronavirus disease 2019 (COVID-19) syndrome, also known as long COVID, is a prolonged illness after the acute phase of COVID-19. Hospitalized patients were known to have persisting symptoms of fatigue, headache, dyspnea, and anosmia. There is a need to describe the characteristics of individuals with post-COVID-19 symptoms in comparison to the baseline characteristics.

**Purpose:**

To investigate the clinical and biochemical characteristics of people who recovered from COVID-19 after 6 months of discharge from the hospital.

**Methods:**

This was a prospective follow-up investigation of hospitalized and discharged COVID-19 patients. Adult patients admitted to King Saud University Medical City, Riyadh, Saudi Arabia, with laboratory-confirmed COVID-19 and discharged were recruited. The baseline demographic information, comorbidities, vital signs and symptoms, laboratory parameters, COVID-19 therapy, and outcomes were collected from the medical records. Blood samples were collected for cytokines estimation. A detailed interview about signs and symptoms was undertaken during the follow-up.

**Results:**

Half of the followed-up people reported experiencing at least one of the COVID-19-related symptoms. The mean blood pressure was found higher in follow-up. People with the symptoms were characterized by low lymphocyte count, lower serum calcium levels, and hyperglycemia compared to people without any post-COVID-19 symptoms. Cytokines IL-8, VEGF, and MCP-1 were higher in people with the most frequent symptoms.

**Conclusion:**

People with post-COVID-19 symptoms were characterized by lower lymphocyte count, lower serum calcium levels, and hyperglycemia compared to people without symptoms. Individuals with the most frequent post-COVID-19 symptoms had higher baseline pro-inflammatory, chemotactic, and angiogenic cytokines.

## Introduction

Coronavirus disease 2019 (COVID-19), caused by the infection with severe acute respiratory syndrome coronavirus 2 (SARS-CoV-2), became a pandemic worldwide within a few months of its appearance in Wuhan, China, in December 2019 (1). After the initial interest in the acute clinical presentation and treatment of COVID-19, the focus has shifted toward understanding the long-term sequelae of COVID-19. Post-acute COVID-19 syndrome, also known as long COVID, is a prolonged illness after the acute phase of COVID-19. It is defined as “the collection of symptoms that develop during or following a confirmed or suspected case of COVID-19, and which continue for more than 28 days” (2). It is often referred to as post-acute sequelae of SARS-CoV-2 infection (PASC). The persisting symptoms after recovery from acute COVID-19 have been reported widely. A systematic review found that 72.5% of patients reported persistence of at least one symptom among patients who were previously hospitalized for COVID-19. Shortness of breath was found to be the most frequently reported symptom during the follow-up (3). Hospitalized patients were known to have persisting symptoms of fatigue, headache, dyspnea, and anosmia. Older age, BMI, and female gender were associated with the long COVID (4). Patients who recovered from the acute COVID-19 within 90 days were less likely to present with persisting symptoms during the follow-up (2). COVID-19 survivors suffer mainly from fatigue or muscle weakness, sleep difficulties, anxiety, and depression (3). A meta-analysis found that 51% of the COVID-19 survivors suffer from fatigue, followed by difficulty in breathing (28%) and anxiety (19%) (5). Disease severity during the hospitalization predicted abnormal chest imaging manifestations after 6 months of discharge (6). Radiological changes persisted in 24% of patients even after 12 months of discharge (6).

Markers of inflammation and coagulopathy continue to be elevated in people who recovered from COVID-19. D-dimer levels at admission predicted the impaired diffusing capacity of the lungs for carbon monoxide (DLCO) after 3 months of discharge (7). A recent study demonstrated that SARS-CoV-2 RNA level in the blood at diagnosis, presence of specific autoantibodies, Epstein-Barr virus viremia, and type 2 diabetes were the risk factors for long COVID (8). Development of long-term COVID-19 sequel correlated with specific changes in immunoglobulins, patient’s age, history of asthma, and number of symptoms during the acute phase of infection (9). Elevated D-dimer levels were observed in 25% of people after 4 months of recovery from COVID-19. Elevated D-dimer level was found more common in people aged more than 50 years (10). Abnormal liver enzymes were seen in a 2-month follow-up study on individuals discharged from COVID-19 hospitalization (11). COVID-19 survivors exhibited lymphocytopenia, elevated inflammatory markers and liver enzymes, dyslipidemia, and hyperuricemia compared to healthy individuals after 3 months of testing negative real-time polymerase chain reaction (RT-PCR) for SARS-CoV-2 (12). Ong et al. (13) reported elevated pro-inflammatory cytokines and pro-angiogenic proteins in the COVID-19 survivors after 6 months. Another study showed higher levels of IL-17 and interleukin-2 (IL-2) in individuals with long COVID (14). COVID-19 follow-up studies mainly emphasized identifying the persisting symptoms, abnormalities in pulmonary function, and radiological changes. Baseline characteristics and continued changes in laboratory parameters are reported in a few studies. There is a need to describe the characteristics of individuals with post-COVID-19 symptoms in comparison to the baseline characteristics. Persistent changes in the laboratory parameters or cytokines may have a role in the post-COVID-19 manifestations. Therefore, the present study was undertaken to investigate the characteristics of clinical and biochemical profiles of COVID-19 recovered people after 6 months of discharge from the hospital to identify the role of clinical and biochemical changes in the post-COVID-19 symptoms.

## Materials and methods

### Study design and participants

This was a prospective follow-up investigation of hospitalized and discharged COVID-19 patients recruited in a previous study (15). Adult patients admitted to King Saud University Medical City, Riyadh, Saudi Arabia, with laboratory-confirmed COVID-19 from June 2020 until November 2020 and discharged alive were recruited for this follow-up study. People were excluded if admission laboratory investigations were unavailable, or patients died, were relocated, were hospitalized, or were not able to visit the follow-up clinic. The baseline demographic information, comorbidities, vital signs and symptoms, laboratory parameters, COVID-19 therapy, and outcomes were collected from the medical records.

### Follow-up assessments

Eligible people were invited to visit the follow-up clinic after 6 months of discharge from the hospital. A detailed interview about persisting signs and symptoms was undertaken during the visit. The participants were asked for the presence of loss of sense of odor, loss of sense of taste, loss of appetite, fever, cough, tiredness, shortness of breath, runny nose, sore throat, body aches, diarrhea, headache, and confusion. They were also asked to report the onset of any new symptoms after the discharge. Blood samples were collected to assess the laboratory parameters. The study protocol was reviewed and approved by the institutional review board of the College of Medicine, King Saud University, Riyadh, Saudi Arabia. Informed consent was obtained from all the study participants.

### Biochemical analysis

A 5 ml of blood sample was collected *via* venipuncture for the Evidence Investigator Cytokine and Growth Factors High-Sensitivity Array measurement during the baseline and at the follow-up. The serum samples were stored frozen in small aliquots at −80°C until analysis. The Evidence Investigator Cytokine High-Sensitivity Array was used to quantitatively measure multiple cytokines from a single sample for detection of IL-2, interleukin-4 (IL-4), interleukin-6 (IL-6), interleukin-8 (IL-8), interleukin-10 (IL-10), vascular endothelial growth factor (VEGF), interferon-γ (IFN-γ), tumor necrosis factor-α (TNF-α), interleukin-1α (IL-1α), interleukin-1β (IL-1β), monocyte chemoattractant protein-1 (MCP-1), and epidermal growth factor (EGF) respectively according to the manufacturer’s instructions (catalog No. EV3623) Randox Laboratories Limited, United Kingdom.

### Data analysis

We compared the characteristics of people at the baseline and the follow-up. The baseline and follow-up characteristics are compared between people with at least one symptom to those who reported no symptoms at the follow-up. Shapiro–Wilk test was performed to determine the normality of distribution of all continuous variables. Continuous data are represented as mean and standard deviation (SD) for normally distributed variables, or median and interquartile range for skewed distributions, and analysis was performed using the independent samples *t*-test, Mann–Whitney U test, or Analysis of Variance (ANOVA), or Proportions are expressed as frequencies and percentages and were compared using the Chi-square test. Data analysis was conducted using the Statistical Package for Social Sciences (SPSS) version 26 (SPSS Inc., Armonk, NY, USA), and a *p*-value of <0.05 was considered statistically significant.

## Results

**FIGURE 1:**
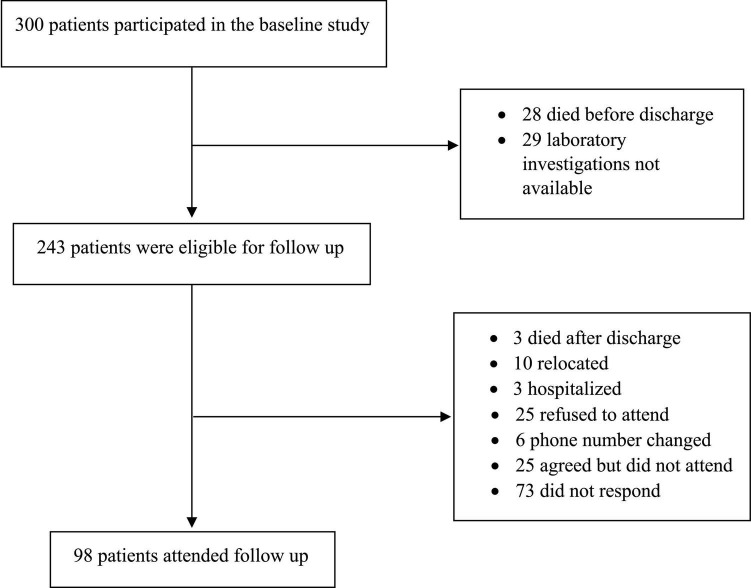
Flow chart for the patient selection process.

Of the 300 patients who participated in the baseline study, 243 eligible for follow-up were called, and 98 attended the follow-up visit (Figure 1). The mean duration of follow-up was 7.02 ± 1.6 months. The demographic and clinical characteristics of people at the follow-up compared to the baseline are given in Table 1. There was no significant change in the BMI between baseline and follow-up periods. The blood pressure, both systolic and diastolic pressures were significantly higher during follow-up. Shortness of breath (22.4%) was the most frequent symptoms reported by the participants during the follow-up. Body aches (20.4%), loss of sense of odor (12.2%), headache (12.2%), and cough (10.2%) were the other dominant symptoms described by the participants. Confusion (9.2%), runny nose (9.2%), tiredness (8.2%), loss of sense of taste (7.1%), loss of appetite (7.1%), sore throat 6.1%), and diarrhea (6.1%) were reported by fewer participants. Fever (4.1%) was the lowest reported symptom in the follow-up. While the proportion of patients who had fever, cough, and shortness of breath was found to be significantly decreased during follow-up (*p* < 0.001, *p* < 0.001, and *p* = 0.028, respectively), whereas loss of sense of odor, runny nose/colds, and confusion were found to be higher during follow-up (*p* = 0.006, *p* = 0.009, and *p* = 0.030, respectively). Symptoms such as loss of appetite, fatigue, sore throat, body aches, diarrhea, and headache were found similar between the baseline and follow-up. Supplementary Table 1 shows the comparison of hematological and biochemical characteristics between baseline and follow-up periods. Most of the abnormally changed parameters found during the baseline period returned to normal values or close to normal values at the follow-up period.

**TABLE 1:** Demographic and clinical characteristics of patients during baseline and at follow-up.

Variables	Baseline	Follow-up	*P*-values
Age, in years	48.87 ± 17.11		
**Gender**			
Males	50 (51.0%)		
Females	48 (49.0%)		
BMI, kg/m^2^	28.11 ± 5.3	28.03 ± 5.8	0.895
SBP, mmHg	124.68 ± 14.9	131.26 ± 15.3	< 0.001*
DBP, mmHg	74.44 ± 11.6	79.51 ± 12.1	0.001*
Loss of sense of odor, yes	2 (2.0%)	12 (12.2%)	0.006*
Loss of sense of taste	2 (2.0%)	7 (7.1%)	0.088
Loss of appetite	9 (9.2%)	7 (7.1%)	0.602
Fever	36 (36.7%)	4 (4.1%)	< 0.001*
Cough	38 (38.8%)	10 (10.2%)	< 0.001*
Tiredness	11 (11.2%)	8 (8.2%)	0.469
Shortness of breath	37 (37.8%)	22 (22.4%)	0.028*
Runny nose/colds	1 (1.0%)	9 (9.2%)	0.009*
Sore throat	9 (9.2%)	6 (6.1%)	0.420
Body aches	13 (13.3%)	20 (20.4%)	0.182
Diarrhea	11 (11.2%)	6 (6.1%)	0.205
Headache	4 (4.1%)	12 (12.2%)	0.488
Confusion	1 (1.0%)	9 (9.2%)	0.030*

*Significant *p*-value between baseline and follow-up. Values are expressed as mean ± SD or numbers and percentage in parenthesis.

In order to assess the characteristics of people who exhibited symptoms during the follow-up, the participants were categorized into those who reported at least one COVID-19-related symptom and those who reported no COVID-19-related symptoms at the follow-up. People with at least one symptom during the follow-up were slightly older (51.34 ± 18.2 vs. 46.44 ± 16.8 years), though the age difference was not significant (*p* = 0.172) (Table 2). The hematological and biochemical characteristics according to the presence or absence of COVID-19-related symptoms are presented in Table 2. Most of the parameters were found to be similar between the people with and without symptoms at the follow-up. People with at least one COVID-19-related symptom had a significantly lower lymphocyte and higher mean corpuscular hemoglobin concentration (MCHC) (*p* = 0.031 and *p* = 0.036, respectively). The serum calcium level was also found to be significantly lower in people with symptoms (*p* = 0.021). They also had significantly higher fasting blood glucose levels at the follow-up when compared to those without any COVID-19-related symptoms (*p* = 0.038). The HbA1c levels were also found higher but not statistically significant (*p* = 0.095).

**TABLE 2:** Six months hematological and biochemical characteristics of patients reporting at least one post-COVID-19 symptom vs. no symptom at follow-up.

Variables	No symptoms (*N* = 57)	With at least one symptom (*N* = 41)	*P*-values
Age, in years	46.44 ± 16.8	51.34 ± 18.2	0.172
BMI	27.89 ± 5.6	28.23 ± 5.8	0.784
SBP, mmHg	120.23 ± 13.7	122.73 ± 15.9	0.405
DBP, mmHg	67.63 ± 9.6	72.10 ± 10.8	0.034
Granulocyte (× 10^9^ L)	3.93 ± 1.9	3.59 ± 1.4	0.560
Lymphocytes (× 10^9^ L)	2.63 ± 0.8	2.35 ± 0.9	0.277
Monocytes (× 10^9^ L)	0.61 ± 0.4	0.61 ± 0.3	0.944
Eosinophils (× 10^9^ L)	0.20 ± 0.2	0.22 ± 0.3	0.102
Basophils (× 10^9^ L)	0.20 ± 0.3	0.22 ± 0.4	0.871
MCV (fl)	85.64 ± 6.4	85.49 ± 8.2	0.925
MCH (pg)	28.60 ± 2.4	29.22 ± 3.3	0.331
MCHC (g/L)	335.37 ± 10.3	341.23 ± 12.6	0.023*
Hemoglobin (g/L)	136.24 ± 17.6	137.84 ± 24.2	0.731
Hematocrit (%)	40.58 ± 4.8	40.34 ± 6.5	0.850
Platelets (× 10^9^ L)	266.16 ± 80.6	253.68 ± 52.2	0.419
MPV (fl)	8.44 ± 0.8	8.46 ± 1.0	0.935
ESR (mm/h)	24.00 (4.0–72.0)	31.00 (8.0–92.0)	0.744
AST (U/L)	19.00 (9.0–68.0)	23.00 (16.0–52.0)	0.330
ALT (U/L)	26.00 (19.0–111.0)	38.00 (21.0–63.0)	0.925
ALP (U/L)	73.00 (41.0–199.0)	73.00 (56.0–162.0)	0.654
GGT (U/L)	33.00 (2.0–88.0)	52.00 (26.0–98.0)	0.238
Direct bilirubin (μmol/L)	2.05 ± 1.6	1.61 ± 0.5	0.401
Total bilirubin (μmol/L)	9.25 ± 4.5	8.68 ± 6.0	0.682
Albumin (g/L)	38.12 ± 4.8	38.96 ± 3.3	0.397
LDH (U/L)	173.38 ± 33.3	171.41 ± 48.8	0.860
Calcium (mmol/L)	2.32 ± 0.2	2.11 ± 0.4	0.034*
Phosphates (mmol/L)	1.19 ± 0.3	1.27 ± 0.3	0.304
Vitamin D (nmol/L)	58.3 ± 30.1	90.9 ± 42.9	0.286
Creatinine (μmol/L)	74.00 (42.0–92.0)	69.29 (48.0–97.0)	0.853
BUN (mmol/L)	4.55 (2.3–5.0)	4.10 (2.2–7.1)	0.600
Microalbuminuria	9.40 (2.0–12.0)	5.70 (1.0–13.2)	0.670
ACR (mg/g)	5.39 (1.01–48.16)	57.03 (0.89–358.92)	0.059
D-dimer (μg/L)	0.69 ± 0.4	0.79 ± 0.3	0.331
Fibrinogen (g/L)	3.45 ± 0.9	3.46 ± 1.0	0.960
INR	0.99 ± 0.3	0.95 ± 0.1	0.416
PT (s)	12.88 ± 1.6	13.32 ± 1.6	0.226
PTT (s)	35.99 ± 7.9	34.15 ± 4.5	0.217
CRP (mg/L)	2.04 (0.17–804.0)	3.54 (0.35–26.4)	0.488
FBS (mmol/L)	5.95 ± 2.0	7.49 ± 3.8	0.038*
HbA1c (%)	5.87 ± 1.6	7.33 ± 2.3	0.095
LDL (mmol/L)	2.93 ± 0.6	2.94 ± 1.2	0.988
HDL (mmol/L)	1.53 ± 1.2	1.46 ± 1.0	0.839
Triglycerides (mmol/L)	1.66 ± 1.1	2.13 ± 1.1	0.064

*Significant *p*-value between no symptoms vs. at least one symptom group. Values are expressed as mean ± SD, or median and interquartile range in the parenthesis or numbers and percentage in the parenthesis.

**FIGURE 2:**
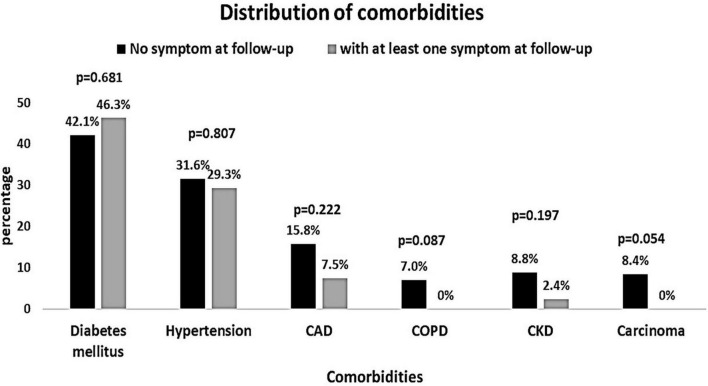
Distribution of comorbidities among patients reporting at least one post-COVID-19 symptom vs. no symptoms at follow-up.

**FIGURE 3:**
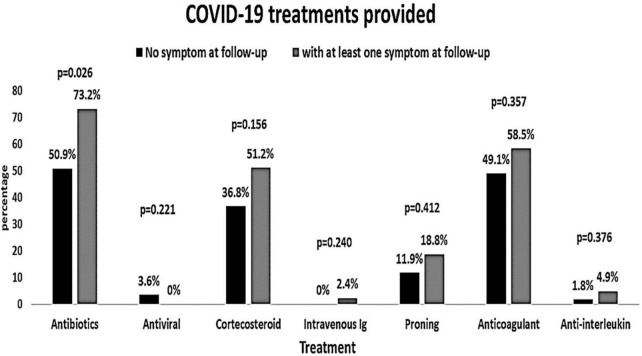
Coronavirus disease 2019 treatments provided to the patients according to the symptoms at the follow-up (at least one post-COVID-19 symptom vs. no symptoms).

The baseline characteristics of the people were compared according to the presence of COVID-19 symptoms at the follow-up (Table 3). People with symptoms at the follow-up had significantly higher AST and microalbuminuria at the baseline (*p* = 0.025 and *p* = 0.009, respectively). All other baseline hematological and biochemical characteristics were found to be similar among those who had at least one COVID-19-related symptom or no symptom at the follow-up. The distribution of comorbidities such as diabetes, hypertension, and coronary heart disease was found to be comparable between people with and without symptoms (Figure 2). The COVID-19 treatment given during the hospitalization is shown in Figure 3. The group of people with at least one COVID-19 symptom had significantly higher cases of people who had high flow nasal cannula during their hospital stay (29.3 vs. 12.3%, *p* = 0.036). All other treatments such as antibiotics, corticosteroids, anticoagulants, non-invasive mechanical ventilation, and proning were found to be similar across people with and without COVID-19-related symptoms. The outcomes such as respiratory failure, ARDS, admission to the intensive care unit, duration of hospitalization, mild COVID-19, and severe COVID-19 were similar between those who reported at least one symptom or no symptoms at the follow-up.

**TABLE 3:** Baseline hematological and biochemical characteristics of patients reporting at least one post-COVID-19 symptom vs. no symptoms at follow-up.

Baseline variables	No symptoms post-COVID-19 (*N* = 57)	With at least 1 symptom post-COVID-19 (*N* = 41)	*P*-values
BMI (kg/m^2^)	29.18 ± 5.7	28.88 ± 9.5	0.852
SBP (mm Hg)	126.30 ± 17.3	125.93 ± 16.1	0.913
DBP (mm Hg)	75.18 ± 12.8	74.93 ± 10.6	0.919
Granulocyte (× 10^9^ L)	4.68 ± 2.4	6.43 ± 2.9	0.260
Lymphocytes (× 10^9^ L)	1.48 ± 0.8	1.27 ± 0.9	0.319
Monocytes (× 10^9^ L)	0.55 ± 0.2	0.55 ± 0.4	0.971
Eosinophils (× 10^9^ L)	0.11 ± 0.3	0.19 ± 0.6	0.549
MCV (fl)	83.07 ± 9.6	85.84 ± 6.4	0.115
MCH (pg)	28.56 ± 3.0	29.21 ± 2.6	0.270
MCHC (g/L)	337.40 ± 11.7	342.49 ± 14.8	0.066
Hemoglobin (g/L)	153.14 ± 19.1	124.12 ± 29.3	0.347
Hematocrit (%)	37.98 ± 4.8	37.40 ± 6.4	0.619
Platelets (× 10^9^ L)	253.43 ± 117.7	271.28 ± 319.3	0.707
MPV (fl)	8.58 ± 1.2	8.55 ± 0.9	0.867
ESR (mm/h)	76.0 (0.51–120)	69.0 (17–99)	0.096
AST (U/L)	48.0 (44–62)	50.5 (18–180)	0.025*
ALT (U/L)	49.0 (36–131)	72.0 (0–179)	0.370
ALP (U/L)	83.0 (59–312)	59.0 (49–354)	0.090
GGT (U/L)	118 (15–555)	106 (0–370)	0.149
Direct bilirubin (μmol/L)	2.42 (1–5)	1.95 (1–10)	0.928
Total bilirubin (μmol/L)	7.70 (4–13)	7.25 (3–17)	0.937
Albumin (g/L)	30.07 ± 5.3	30.97 ± 4.6	0.416
LDH (U/L)	367 (107–657)	386 (144–955)	0.641
Calcium (mmol/L)	2.22 ± 0.2	2.12 ± 0.3	0.107
Phosphates (mmol/L)	1.07 (0.8–1.51)	1.17 (0.57–2.05)	0.377
Vitamin D (nmol/L)	23.75 (15–76)	34.90 (18–117)	0.545
Creatinine (μmol/L)	82.0 (45–733)	82.0 (54–145)	0.315
BUN (mmol/L)	6.0 (2–30)	4.60 (2–10)	0.777
Microalbuminuria	44.89 ± 64.3	106.51 ± 144.1	0.009*
ACR (mg/g)	36.6 (3.72–1,179.42)	161.2 (20.70–311.87)	0.341
D-Dimer (μg/L)	1.46 ± 1.3	1.21 ± 0.9	0.411
Fibrinogen (g/L)	5.05 ± 2.2	6.39 ± 2.1	0.072
INR	1.06 ± 0.2	1.09 ± 0.2	0.348
PT (s)	14.48 ± 4.6	14.45 ± 3.0	0.951
PTT (s)	38.42 ± 5.4	41.09 ± 9.3	0.273
CRP (mg/L)	86.50 (2–135)	66.4 (4–164)	0.401
FBS (mmol/L)	7.89 ± 4.7	8.54 ± 5.0	0.662
HbA1c (%)	8.62 ± 3.7	11.89 ± 1.2	0.120
LDL (mmol/L)	2.40 ± 1.4	1.95 ± 0.9	0.235
HDL (mmol/L)	1.01 ± 0.4	1.02 ± 0.6	0.841
Triglycerides (mmol/L)	1.84 ± 0.9	1.90 ± 0.8	0.329

*Significant *p*-value between no symptoms vs. at least one symptom group. Values are expressed as mean ± SD, or median and interquartile range in the parenthesis or numbers and percentage in the parenthesis.

The comparative characteristics of people who had symptoms and those who had no symptoms during the hospitalization at the baseline are shown in Supplementary Table 2. There is no remarkable difference between patients with and without symptoms during the hospitalization except for serum AST and fibrinogen levels which were found significantly higher in people who had symptoms in the baseline (*p* = 0.042 and *p* = 0.003, respectively). The mean age of the participants who had symptoms was higher (50.2 ± 17.6 years) than those who did not have any symptoms (43.5 ± 14.3 years) during the baseline. However, this was not statistically significant (*p* = 0.065). Similarly, blood glucose and HbA1c levels were also seen high in people with symptoms but it was not statistically significant (*p* = 0.078 and *p* = 0.174, respectively). All other hematological and biochemical parameters were similar between people with and without symptoms during the baseline period.

The cytokines profile of patients at baseline and follow-up were compared and is shown in Table 4. Most of the cytokine levels were found lowered at the follow-up. The levels of IL-6, IL-8, IL-10, VEGF, IFN-γ, TNF-α, IL-1β, and EGF were significantly lower during the follow-up. The IL-4 level was significantly increased (*p* = 0.001) and the levels of IL-2 and MCP-1 did not change (*p* = 0.525 and *p* = 0.380, respectively). There was no significant difference in cytokines between individuals with and without symptoms. Subsequently, we compared the cytokine levels of people with the most frequent symptoms individually at the follow-up to people without any symptoms. Baseline IL-10 (*p* = 0.008) and VEGF (*p* = 0.024) levels were found higher and baseline IL-4 (*p* = 0.010) was lower in people who reported shortness of breath (Supplementary Figure 1). IL-2 and MCP-1 were elevated both at baseline and at follow-up whereas IL-1β and EGF were lower at baseline in individuals with loss of sense of odor when compared to people without any symptoms. In addition, IFN-γ and IL-1α were found higher during the baseline only (Supplementary Figure 2). However, all these changes were not statistically significant. The group of people reporting myalgia had a slightly lower MCP-1 at both baseline and follow-up when compared to people without any symptoms (Supplementary Figure 3). Patients with headache as one of the symptoms at the follow-up had higher IL-2 at baseline and follow-up. They also had higher IL-10, IFN-γ, and MCP-1 at baseline and higher IL-4 at follow-up when compared to individuals without any symptoms (Supplementary Figure 4). These differences were also not statistically different. In individuals with cough, IL-6, IL-8, IL-10, IFN-γ, and MCP-1 were higher at baseline (Supplementary Figure 5). IL-8 (*p* = 0.010), IL-10 (*p* = 0.012), and MCP-1 (*p* = 0.008) were statistically higher in individuals with cough. When the most frequent five symptoms were combined and compared with patients who had no symptoms, levels of IL-8 (*p* = 0.023) and VEGF (*p* = 0.041) were higher and IL-4 was slightly lower at baseline, and MCP-1 was higher at both baseline and at follow-up in people with symptoms (Supplementary Figure 6). Many of the changes in the cytokines were not statistically significant owing to the smaller sample size, nevertheless, these differences indicate the trend of change in the cytokine levels.

**TABLE 4:** Cytokine levels of the patients during baseline and follow-up and according to the presence of symptoms at the follow-up.

Cytokines (pg/ml)	Baseline (median, IQR)	Follow-up (median, IQR)	*P*-value*	No symptom at follow-up (median, IQR) *N* = 57	With at least one symptom at follow-up (median, IQR) *N* = 41	*P*-value
IL-2	0.00 (0–19.3)	0.00 (0–29.23)	0.525	0.00 (0–29.30)	0.00 (0–22.55)	0.444
IL-4	1.10 (0–9.02)	1.21 (0–13.89)	0.001*	1.22 (0–13.89)	1.19 (0.49–2.13)	0.913
IL-6	7.19 (0.39–290.95)	1.72 (0.35–95.23)	< 0.001*	1.74 (0.50–6.95)	1.69 (0.35–95.23)	0.346
IL-8	18.40 (1.88–797)	8.41 (1.59–38.60)	< 0.001*	8.05 (1.59–38.60)	8.71 (2.70–30.30)	0.135
IL-10	1.11 (0–73.36)	0.30 (0–13.12)	< 0.001*	0.27 (0.17–13.12)	0.31 (0–2.51)	0.148
VEGF	191.12 (0–848.36)	108.71 (4.22–587.17)	< 0.001*	106.23 (4.22–587.17)	114.72 (13.68–557)	0.691
IFN-γ	0.44 (0–46.79)	0.25 (0–1.84)	< 0.001*	0.26 (0–1.24)	0.24 (0–1.84)	0.441
TNF-α	5.73 (2.11–24.74)	4.24 (1.19–12.13)	< 0.001*	4.05 (1.19–12.13)	4.61 (2.07–8.61)	0.389
IL-1α	0.00 (0–0.77)	0.04 (0–0.73)	0.217	0.00 (0–0.52)	0.08 (0–0.73)	0.597
IL-1β	0.75 (0–9.31)	0.68 (0–12.03)	0.029*	0.68 (0–12.03)	0.68 (0–4.11)	0.809
MCP-1	148.38 (14.89–697.0)	142.70 (24.1–447.14)	0.380	144.62 (30.37–447.14)	137.76 (24.10–426.96)	0.609
EGF	107.96 (0.94–392.49)	35.72 (2.38–209.08)	< 0.001*	34.97 (3.27–132.19)	44.05 (2.38–209.08)	0.770

*Significant *p*-value between baseline vs. follow-up. Values are expressed as median and interquartile ranges in the parenthesis. IL-2, interleukin-2; IL-4, interleukin-4; IL-6, interleukin-6; IL-8, interleukin-8; IL-10, interleukin-10; VEGF, vascular endothelial growth factor; IFN-γ, interferon γ; TNF-α, tumor necrosis factor α; IL-1α, interleukin-1α; 1β, interleukin-1β; MCP-1, monocyte chemoattractant protein-1; EGF, epidermal growth factor.

## Discussion

The follow-up characteristics of people who were hospitalized for acute COVID-19 reported in this study show that almost half of them experience post-COVID-19 related symptoms. A total of 41.8% of participants who attended the follow-up visit reported that they experienced at least one COVID-19-related symptom. This is comparable to the prevalence of post-acute COVID-19 symptoms reported in other studies. A meta-analysis reported a prevalence of 45.9% for post-COVID-19 symptoms at >90 days follow-up and 45% at 6–9 months follow-up (16). In another study, 44.2% of the people experienced persistent COVID-19-related symptoms after 1 year (17). The most common symptom during the follow-up in our study was dyspnea (22.4%), followed by myalgia (20.4%). Fatigue (37%) and dyspnea (21%) were reported to be the most common symptoms of the post-COVID-19 at 6–9 months follow-up (18). The comparison of hematological and biochemical characteristics of the people between baseline and follow-up periods indicates that most of the parameters significantly recovered to normal range or closer to normal range. In a study on COVID-19 survivors, persistent abnormalities in many biochemical parameters have been reported after 3 months of acute COVID-19 infection (12). However, they compared the COVID-19 survivors with healthy controls. Our results show that there is a significant recovery of biochemical parameters in people after 6 months of acute COVID-19 infection when compared to their baseline. Since almost half of our study population showed persistent COVID-19-related symptoms, we decided to compare the biochemical parameters of people with at least one symptom against people without any COVID-19-related symptoms.

Even though most of the hematological and biochemical parameters are comparable between people who had symptoms and who had no symptoms at the follow-up, the lymphocytes, MCHC, serum calcium, and blood glucose levels significantly differed between them. Lymphopenia is commonly seen in COVID-19 patients. It is reported to be significantly lower in people with moderate COVID-19 compared to asymptomatic people (19). Low lymphocyte count was shown to be an indicator of disease severity in COVID-19 cases (20). Lymphocytes are crucial in regulating cellular immunity, and a significant reduction in COVID-19 patients is an indication of their destruction by the virus (21). Hence, critically ill COVID-19 patients are usually characterized by a drastic reduction in the lymphocyte count. Our results show that symptomatic people have significantly lower lymphocyte count than asymptomatic people after 6 months of acute COVID-19 infection. A study that followed mild to moderate COVID-19 survivors reported lower lymphocyte count irrespective of the symptoms after 90 days of the acute infection when compared to healthy subjects (12). When we looked into the baseline lymphocyte count, the symptomatic people had only a slightly lesser lymphocyte count, and it is not statistically significant. Further, the number of severe COVID-19 cases was similar between those with and without symptoms at the follow-up. Therefore, it is unlikely that the severity of COVID-19 during the baseline period could have led to the low lymphocyte count at the follow-up. A significantly higher level of MCHC was observed in people with symptoms at the follow-up. Usually, lower levels of MCHC are reported to be associated with disease severity and mortality in COVID-19 (22). But in a previous study, significantly higher MCHC levels are found in COVID-19 survivors regardless of the post-COVID-19 symptoms compared to healthy subjects (12). In our study, there was no change in the MCHC levels between baseline and follow-up. But when the baseline MCHC levels are compared between people with and without symptoms at follow-up, it was slightly higher in people with symptoms, although statistically not significant. Therefore, it may be also possible that this group of people had a higher baseline MCHC that continued in the follow-up.

Severe acute respiratory syndrome coronavirus 2 infection can lead to a lowering of serum calcium levels and the severity of COVID-19 was known to be associated with hypocalcemia. Previous studies have shown that hypocalcemia correlated strongly with hyper-inflammatory response and coagulopathy (23, 24). Serum calcium level in the current study population is found to be significantly lower in people with symptoms during the follow-up. At baseline, the serum calcium levels were not significantly lower in people with symptomatic COVID-19 infection or people with post-COVID-19-related symptoms. The difference between baseline and follow-up serum calcium levels is also found to be minimal. Serum calcium levels were lower only in people with symptoms at the follow-up. The hypocalcemia observed during the acute COVID-19 was reported to be independent of vitamin D deficiency, albumin levels, and disease severity (25). In our study too, the serum vitamin D and albumin levels at the follow-up are comparable between people with and without symptoms. It may be possible that the lower serum calcium level is also linked to the post-COVID-19-related symptoms. There are reports of hypoparathyroidism induced by COVID-19 infection (26). However, we do not know how many of our patients developed hypoparathyroidism and its influence on serum calcium levels.

The other characteristic of people with post-COVID-19-related symptoms in our study is hyperglycemia. Fasting blood glucose levels are significantly higher in people with symptoms at the follow-up when compared to people without any symptoms. Moreover, the fasting blood glucose and HbA1c levels are in the diabetic range in people with symptoms. The baseline glycemic parameters are very high when compared to the follow-up, indicating the presence of glycometabolic disturbance during the acute phase of COVID-19. The hyperglycemia had decreased post-discharge in many participants but only marginally in those who had persistent symptoms after the discharge. In addition, people with at least one COVID-19-related symptom during the baseline period had high mean blood glucose and HbA1c levels at baseline. This shows a possible relationship between hyperglycemic conditions and the presence of COVID-19-related symptoms. Corticosteroid therapy is one of the major contributors to uncontrolled blood glucose levels. It is also possible that people who needed corticosteroid therapy during the hospitalization experienced more post-COVID-19 symptoms. The number of people who received corticosteroid treatment was found slightly higher in people with symptoms.

Glycometabolic abnormalities caused by acute COVID-19 infection are known to persist even after recovery (27). COVID-19 infection has increased the cases of incident diabetes after the acute phase of infection (28). We did not collect information about new incident cases of diabetes post-COVID-19 from the study population. Nevertheless, the glycemic data shows that abnormal blood glucose levels in the acute phase of COVID-19 infection continued even after the recovery in most people with persistent post-COVID-19-related symptoms. Further investigation in a larger population with post-COVID-19 symptoms and glycemic abnormalities may be needed to confirm this finding. The other baseline parameters of the people with and without symptoms at the follow-up were found to be mostly similar between them. Only higher serum AST levels and microalbuminuria were observed among people with symptoms. We also compared the baseline variables of people with and without symptoms during the baseline period. Interestingly, the baseline serum AST levels were significantly higher in people with symptoms at the baseline as well.

The cytokine profile showed specific changes with symptoms when compared to people without any symptoms at the follow-up. People who continued to experience shortness of breath had higher baseline VEGF levels. Shortness of breath triggers hypoxia in the patients. VEGF is reported to be upregulated in hypoxic conditions and is involved in mediating acute lung injury (29). It is possible that most of the patients who had persistent dyspnea were likely to have had severe hypoxic conditions during the acute phase of COVID-19 infection. A previous study reported elevated VEGF in COVID-19 patients on arrival at the hospital (30). Individuals with loss of sense of odor had higher IL-2, IFN-γ, IL-1α, and MCP-1 at the baseline. IL-2 is a pro-inflammatory cytokine that stimulates the activation of T-cells to produce inflammatory cytokines. A higher IL-2 level is an indication of ongoing immune response (31). In COVID-19, IL-2 levels are known to be associated with mild and asymptomatic conditions (32). However, in our study the IL-2 levels were seen elevated both at baseline and at follow-up, indicating the ongoing immune reaction. Baseline high IFN-γ indicates a good immune response to the viral infection. In a prospective cohort study, the high IFN-γ was associated with poor outcomes in COVID-19 patients (33). The level of MCP-1 was found elevated both at baseline and follow-up. A high level of MCP-1 has been reported to be present in the early phase of COVID-19 (13). However, in our study, the MCP-1 level appeared to increase during the follow-up.

Individuals who reported myalgia at follow-up had lower levels of MCP-1 both at the baseline and at the follow-up period. Since the increased MCP-1 levels were associated with worst outcomes in COVID-19 patients (34), the continued lower levels of MCP-1 could be an indication of milder acute COVID-19 and a milder post-COVID sequel in those individuals. Headache is the third most frequent symptom reported by the participants. They showed a high baseline of IL-2, IL-10, IFN-γ, and MCP-1. In the follow-up only IL-2 and IL-4 were found to be higher. Higher levels of IL-2, IL-10, IFN-γ, and MCP-1 are indicators of mild COVID-19 as discussed earlier. IL-10 is a potent anti-inflammatory cytokine and is known for its immunosuppressive effects. It is thought that the increase in IL-10 is compensatory in an attempt to dampen the inflammation and prevent further damage (35). Another anti-inflammatory cytokine IL-4 was found higher during the follow-up in people with headaches. In people with cough, pro-inflammatory cytokines IL-6, IL-8, anti-inflammatory cytokine IL-10, and chemotactic cytokine MCP-1 were higher at baseline. IL-8 a pro-inflammatory cytokine with the neutrophil chemotactic function was found higher in patients with the most frequent symptoms at the follow-up. IL-8 has been portrayed as a biomarker of hyper-inflammatory conditions in acute respiratory distress syndrome (36). It is also reported to be angiogenic. When all the most frequent five symptoms were combined baseline IL-8, VEGF, and MCP-1 were higher. Common inflammatory markers such as TNF-α and IL-6 are not significantly different between people with and without symptoms during the follow-up. Given that the levels of TNF-α and IL-6 were in the normal range, it appears that there was no active inflammation in people during the follow-up. Putting all together, the individuals with the most frequent symptoms at the follow-up were characterized by a higher baseline pro-inflammatory, chemotactic and angiogenic cytokines. It may be noted that many of the cytokine levels are not statistically different and the changes are only an indication of the trend. Therefore, these findings need to be interpreted cautiously.

The present study shows that the effect of COVID-19 infection goes beyond the hospitalization period. Almost half of the followed-up people reported experiencing at least one of the COVID-19-related symptoms. The mean blood pressure was found higher in follow-up. People with the symptoms were characterized by low lymphocyte count, lower serum calcium levels, and hyperglycemia compared to people without any post-COVID-19 symptoms. These changes were not seen in people with symptoms during the baseline period except for hyperglycemia. Corticosteroid therapy might have aggravated the hyperglycemia, triggered initially by the acute COVID-19 infection. It appears that the presence of hyperglycemia may have an association with COVID-19-related symptoms. The relationship of persistent post-COVID-19 symptoms with hyperglycemia needs further investigation. Other differences like low lymphocyte count and lower serum calcium are also usually associated with acute COVID-19 infection. Their relationship with the post-COVID-19 conditions is not clear. As the distribution of comorbidities and severe COVID-19 cases were similar between the groups, it seems unlikely that the history of severe COVID-19 could have led to the persistent post-COVID-19 related symptoms. Pro-inflammatory, chemotactic, and angiogenic cytokines were found higher at baseline in people with the most frequent symptoms when compared to those without symptoms. It needs further research in a larger population to confirm these findings and to identify the pathophysiology behind the continued COVID-19-related symptoms. Interestingly, having a vaccination for COVID-19 reported to decrease the chances of post-COVID-19 symptoms. Moreover, there was a sustained improvement in the post-COVID-19 symptoms after the booster dose (37). COVID-19 vaccines could be a potential treatment for post-COVID-19 conditions.

A major strength of our study is that we investigated the follow-up clinical laboratory parameters and compared them to the baseline. As many participants reported post-COVID-19 related symptoms, we explored the biochemical and inflammatory characteristics of people who continued to experience the symptoms in comparison to those who did not report any symptoms during the follow-up. In addition, the cytokine profile was assessed in the baseline and follow-up. Limitations of this study include a smaller study population and high lost to follow-up. The high number of lost to follow-up might have led to an underestimation of the prevalence of post-COVID-19-related symptoms. Another limitation is we did not exclude any asymptomatic reinfections at the follow-up. The baseline data may not reflect the actual disease severity as many patients would have been in an early stage of the disease then. The study was conducted on patients from a single center and thus not generalizable.

## Conclusion

The clinical and biochemical characteristics of people with post-COVID-19-related symptoms were described in this study. Almost half of the study population reported experiencing at least one COVID-19-related symptom after 6 months of discharge from hospitalization due to COVID-19. People with symptoms at the follow-up were characterized by lower lymphocyte count, lower serum calcium levels, and hyperglycemia compared to people without any symptoms. Individuals with the most frequent post-COVID-19 symptoms had higher baseline pro-inflammatory, chemotactic and angiogenic cytokines. Further studies are needed to identify the pathophysiology behind the continued COVID-19-related symptoms.

## Data availability statement

The original contributions presented in this study are included in the article/Supplementary material, further inquiries can be directed to the corresponding author.

## Ethics statement

The studies involving human participants were reviewed and approved by the Institutional Review Board, College of Medicine, King Saud University. The patients/participants provided their written informed consent to participate in this study.

## Author contributions

AA designed the study, involved in overall supervision, data analysis, interpretation of results, and reviewing the manuscript. MR conceived the idea, designed the study, involved in the study coordination, data analysis, interpretation of results, and wrote the manuscript. NA, KB, HA, IA, MAa, and MFA involved in patient recruitment, patient follow-up, interpretation of results, and review of the manuscript. MAk, MAz, SA, and KA involved in patient recruitment, patient follow-up, interpretation of results, and review of the manuscript. KS conduced the biochemical analysis, involved in the data analysis, interpretation of results, and review of the manuscript. AY involved in the coordination of the study, the patient follow-up, and the review of the manuscript. AI involved in the data analysis, interpretation of results, and review of the manuscript. SN involved in the biochemical analysis, interpretation of results, and review of the manuscript. All authors contributed to the article and approved the submitted version.

## Supplementary material

Supplementary Table 1 Hematological and biochemical characteristics of patients during baseline and at follow-up.

Supplementary Table 2 Follow-up hematological and biochemical characteristics of patients who had at least one COVID-19-related symptom vs. no symptoms at baseline.

Supplementary Figure 1 Baseline and follow-up cytokine levels of patients who had shortness of breath (dyspnea) vs. people without any symptoms at the follow-up.

Supplementary Figure 2 Baseline and follow-up cytokine levels of patients who had loss of sense of odor vs. people without any symptoms at the follow-up.

Supplementary Figure 3 Baseline and follow-up cytokine levels of patients who had myalgia vs. people without any symptoms at the follow-up.

Supplementary Figure 4 Baseline and follow-up cytokine levels of patients who had headache vs. people without any symptoms at the follow-up.

Supplementary Figure 5 Baseline and follow-up cytokine levels of patients who had cough vs. people without any symptoms at the follow-up.

Supplementary Figure 6 Baseline and follow-up cytokine levels of patients who had either dyspnea, loss of sense of odor, myalgia, headache, or cough vs. people without any symptoms at the follow-up.
