# Supplementary material for: Clinical and biochemical characteristics of people experiencing post-coronavirus disease 2019-related symptoms: A prospective follow-up investigation
Source: Front Med (Lausanne). 2022 Dec 6;9:1067082. doi: 10.3389/fmed.2022.1067082 (PMC9763306; doi:10.3389/fmed.2022.1067082)
Supplement: Supplementary Table 1 — Hematological and biochemical characteristics of patients during baseline and at follow-up. [file Table_1.DOCX]

**Supplementary Table 1. Hematological and biochemical characteristics of patients during baseline and at follow up**

| **Variables** | **Baseline (n=98)** | **Follow up (n=98)** | **P values** |
| --- | --- | --- | --- |
| Granulocyte, (x 10^9^L) | 5.75 (2.1-9.4) | 2.50 (1.4-8.3) | 0.213 |
| Lymphocytes, (x 10^9^L) | 1.35 (1.10-1.60) | 2.30 (0.6-3.8) | <0.001* |
| Monocytes, (x 10^9^L) | 0.95 (0.7-1.2) | 0.5 (0.3-2.0) | 0.736 |
| Eosinophils, (x 10^9^L) | 0.26 (0.21-0.30) | 0.20 (0-1.3) | 0.007* |
| Basophils, (x 10^9^L) | 0.05 (0-0.10) | 0.1 (1.3) | 0.425 |
| MCV, (fL) | 84.70 ± 6.6 | 85.57 ± 7.2 | 0.379 |
| MCH, (pg) | 28.84 ± 2.81 | 28.88 ± 2.8 | 0.921 |
| MCHC, (g/L) | 340.04 ± 13.0 | 338.01 ± 11.6 | 0.252 |
| Hemoglobin, (g/L) | 127.37 ± 19.4 | 136.96 ± 20.7 | 0.001* |
| Hematocrit, (%) | 37.74 ± 5.4 | 40.47 ± 5.6 | 0.001* |
| Platelets, (x 10^9^L) | 245.47 ± 106.9 | 260.52 ± 69.1 | 0.243 |
| MPV, (fL) | 8.59 ± 1.1 | 8.45 ± 0.9 | 0.334 |
| ESR, (mm/hr) | 69.50 (68-71) | 24.0 (4-98) | 0.001* |
| AST, (U/L) | 24.0 (20-28) | 15.0 (7-68) | <0.001* |
| ALT, (U/L) | 9.0 (0-18) | 26.0 (15-111) | <0.001* |
| ALP, (U/L) | 58.0 (56-60) | 74.0 (41-196) | 0.051 |
| GGT, (U/L) | 55.0 (15-206) | 21.5 (10-70) | 0.035* |
| Direct bilirubin, (μmol/L) | 1.50 (0-11) | 1.61 (0.47-3.51) | 0.037* |
| Total bilirubin, (μmol/L) | 8.26 (4-18) | 7.08 (4.41-12.35) | 0.734 |
| Albumin, (g/L) | 30.7 ± 4.8 | 38.5 ± 4.2 | <0.001* |
| LDH, (U/L) | 332.0 (107-955) | 187.0 (155-219) | <0.001* |
| Calcium, (mmol/L) | 2.12 (0-13) | 1.89 (1.55-2.22) | 0.456 |
| Phosphates, (mmol/L) | 1.21 ± 0.7 | 1.24 ± 0.3 | 0.830 |
| Vitamin D, (nmol/L) | 26.5 (18-76) | 48.45 (33.9-102.3) | 0.317 |
| Creatinine, (μmol/L) | 87.0 (40-139) | 109 (90-874) | <0.001* |
| BUN, (mmol/L) | 4.65 (2-96) | 40.0 (2.2-103) | <0.001* |
| Microalbuminuria | 12.5 (3.45-1179) | 4.4 (1-13.2) | 0.002* |
| ACR, (mg/g) | 46.25 (3.45-1179) | 9.93 (0.3-358.92) | 0.002* |
| Troponin | 16.57 ± 40.57 | 1.45 ± 0.52 | <0.001 |
| CK | 240.60 ± 384.52 | 113.50 ± 63.95 | 0.0015 |
| D-Dimer, (μg/L) | 0.84 (1-2) | 0.72 (0.27-2.48) | 0.009* |
| Fibrinogen, (g/L) | 5.78 ± 2.2 | 3.46 ± 0.9 | <0.001* |
| INR | 1.07 ± 0.2 | 0.97 ± 0.3 | 0.001* |
| PT, (s) | 14.59 ± 1.6 | 13.06 ± 1.6 | <0.001* |
| PTT, (s) | 40.09 ± 6.2 | 35.23 ± 6.7 | <0.001* |
| CRP, (mg/L) | 68.20 (2-164) | 2.26 (0.17-26.4) | <0.001* |
| FBS, (mmol/L) | 8.61 ± 5.1 | 6.57 ± 2.9 | 0.001* |
| HbA1c, (%) | 7.61 ± 2.6 | 6.66 ± 2.1 | 0.0053* |
| LDL, (mmol/L) | 2.25 ± 1.2 | 2.93 ± 0.8 | <0.001* |
| HDL, (mmol/L) | 0.87 (0.27-1.19)** | 1.23 (0.57-7.53)** | 0.173 |
| Triglycerides, (mmol/L) | 1.68 (1.02-3.08) | 1.42 (0.46-3.59) | 0.877 |

* Significant *p*-value between baseline Vs. follow up. Values are expressed as mean ± standard deviation, or median and interquartile range in the parenthesis or numbers and percentage in the parenthesis.

**Supplementary Table 2. Follow up hematological and biochemical characteristics of patients who had at least one COVID-19-related symptom Vs. no symptoms at baseline**

| **Baseline variables** | **Baseline symptoms** | | **P values** |
| --- | --- | --- | --- |
|  | **No symptom (n=19)** | **With at least 1 symptom (n=79** |  |
| Age, (years) | 43.5 ± 14.3 | 50.2 ± 17.6 | 0.130 |
| BMI, (kg/m^2^) | 28.1 ± 3.3 | 29.3 ± 8.1 | 0.556 |
| SBP, mmHg | 116.76 ± 12.3 | 124.12 ± 15.3 | 0.014 |
| DBP, mmHg | 68.40 ± 9.6 | 70.20 ± 10.8 | 0.402 |
| Granulocyte, (x 10^9^L) | 3.3 ± 1.5 | 6.1 ± 1.9 | 0.355 |
| Lymphocytes, (x 10^9^L) | 1.7 ± 1.0 | 1.3 ± 0.8 | 0.154 |
| Monocytes, (x 10^9^L) | 0.6 ± 0.2 | 0.6 ± 0.3 | 0.830 |
| Eosinophils, (x 10^9^L) | 0.3 ± 0.6 | 0.1 ± 0.5 | 0.407 |
| MCV, (fL) | 83.5 ± 5.3 | 84.4 ± 9.0 | 0.701 |
| MCH, (pg) | 27.9 ± 2.1 | 29.1 ± 2.9 | 0.156 |
| MCHC, (g/L) | 334.5 ± 8.6 | 340.5 ± 13.8 | 0.097 |
| Hemoglobin, (g/L) | 125.3 ± 17.3 | 144.1 ± 19.3 | 0.646 |
| Hematocrit, (%) | 37.7 ± 4.9 | 37.7 ± 5.6 | 0.984 |
| Platelets, (x 10^9^L) | 271.1 ± 84.2 | 259 ± 54.0 | 0.849 |
| MPV, (fL) | 8.5 ± 0.8 | 8.6 ± 1.1 | 0.833 |
| ESR, (mm/hr) | 40 (17-63) | 71 (0.51-120) | 0.094 |
| AST, (U/L) | 62 (45-79) | 48 (18-180) | 0.042* |
| ALT, (U/L) | 155 (131-179) | 49 (0-111) | 0.264 |
| ALP, (U/L) | 71.5 (59-84) | 74 (49-354) | 0.154 |
| GGT, (U/L) | 62 (18-101) | 50 (4-675) | 0.261 |
| Direct bilirubin, (μmol/L) | 2.0 ± 1.6 | 3.2 ± 2.9 | 0.159 |
| Total bilirubin, (μmol/L) | 8.6 ± 5.0 | 12.23 ± 11.1 | 0.257 |
| Albumin, (g/L) | 30.4 ± 5.3 | 30.5 ± 5.0 | 0.972 |
| LDH, (U/L) | 306.9 ± 185.7 | 414.6 ± 297.6 | 0.253 |
| Calcium, (mmol/L) | 2.2 ± 0.2 | 2.2 ± 0.2 | 0.383 |
| Phosphates, (mmol/L) | 1.2 ± 0.2 | 1.3 ± 1.0 | 0.699 |
| Vitamin D, (nmol/L) | 42.6 (18-67) | 33.65 (19-117) | 0.777 |
| Creatinine, (μmol/L) | 78 (69-92) | 78.5 (4-145) | 0.340 |
| BUN, (mmol/L) | 4.4 (3-5) | 4.8 (2-96) | 0.091 |
| Microalbuminuria | 12 (4-334) | 15 (1-462) | 0.371 |
| ACR, (mg/g) | 115.2 (5.7-205.5) | 46.4 (6.2-1179.4) | 0.848 |
| D-Dimer, (μg/L) | 1.7 ± 1.6 | 1.3 ± 1.0 | 0.257 |
| Fibrinogen, (g/L) | 4.1 ± 1.7 | 6.4 ± 2.1 | 0.003* |
| INR | 1.1 ± 0.1 | 1.1 ± 0.2 | 0.649 |
| PT, (s) | 14.6 ± 1.3 | 14.5 ± 2.4 | 0.870 |
| PTT, (s) | 37.2 ± 5.4 | 40.1 ± 7.7 | 0.232 |
| CRP, (mg/L) | 63.25 (0-171) | 86.7 (3-270) | 0.347 |
| FBS, (mmol/L) | 5.5 ± 1.9 | 8.7 ± 5.0 | 0.078 |
| HbA1c, (%) | 5.9 ± 0.3 | 8.2 ± 3.2 | 0.174 |
| LDL, (mmol/L) | 2.2 ± 1.4 | 2.2 ± 1.2 | 0.941 |
| HDL, (mmol/L) | 0.9 ± 0.3 | 1.0 ± 0.6 | 0.604 |
| Triglycerides, (mmol/L) | 1.7 ± 0.8 | 1.9 ± 1.0 | 0.499 |

* Significant *p*-value between no symptoms Vs. at least one symptom group. Values are expressed as mean ± standard deviation, or median and interquartile range in the parenthesis or numbers and percentage in the parenthesis.
