# Supplementary figures and images for: Clinical and biochemical characteristics of people experiencing post-coronavirus disease 2019-related symptoms: A prospective follow-up investigation
Source: Front Med (Lausanne). 2022 Dec 6;9:1067082. doi: 10.3389/fmed.2022.1067082 (PMC9763306; doi:10.3389/fmed.2022.1067082)

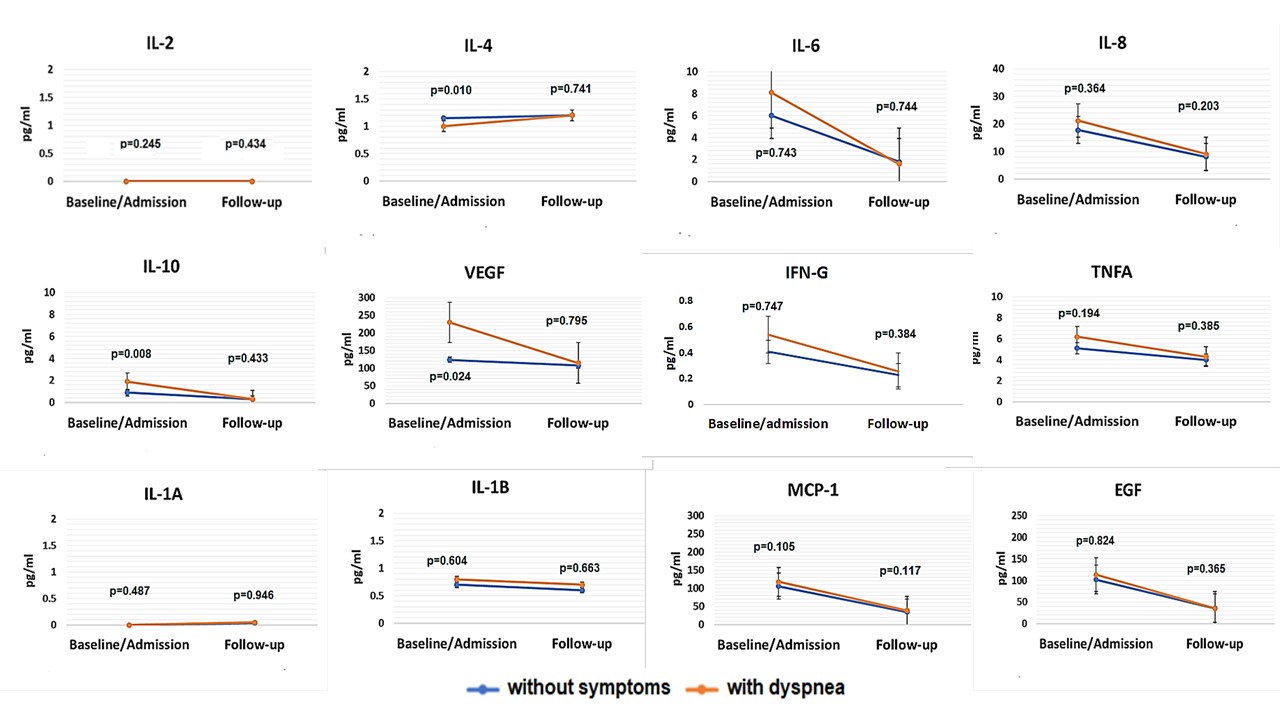

Supplement: Supplementary Figure 1 — Baseline and follow-up cytokine levels of patients who had shortness of breath (dyspnea) vs. people without any symptoms at the follow-up. [file Image_1.JPEG]

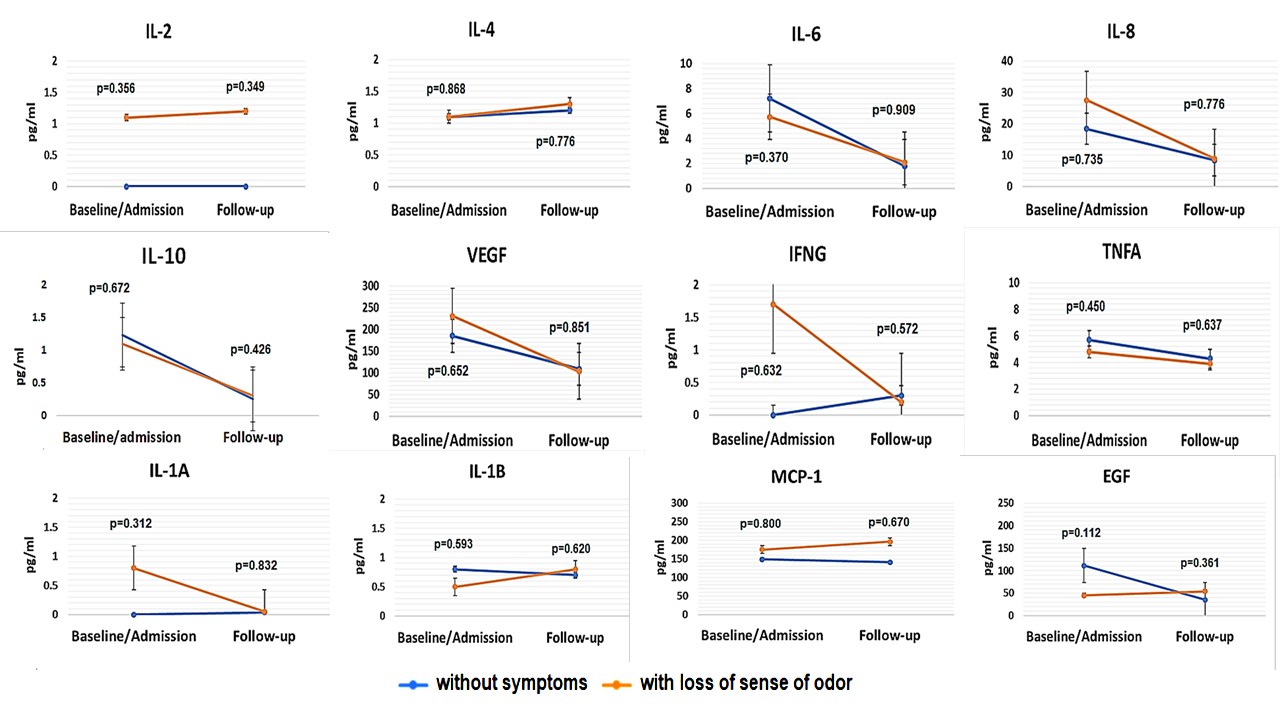

Supplement: Supplementary Figure 2 — Baseline and follow-up cytokine levels of patients who had loss of sense of odor vs. people without any symptoms at the follow-up. [file Image_2.JPEG]

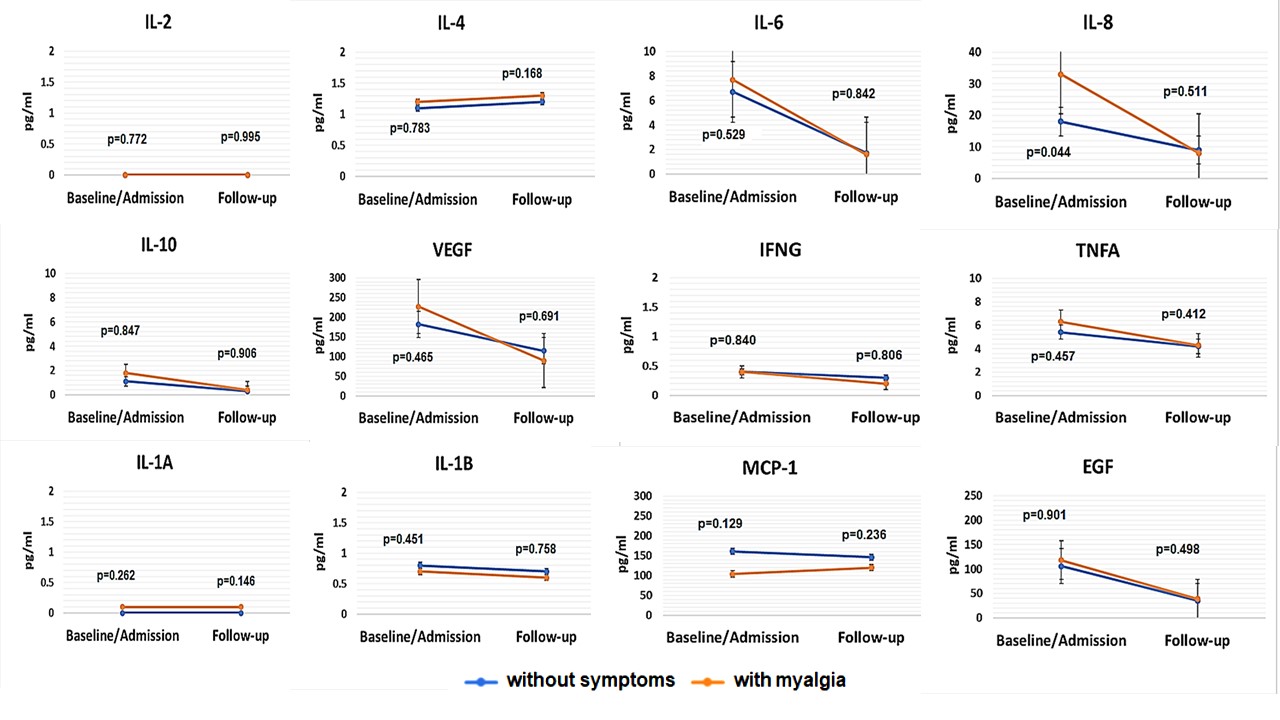

Supplement: Supplementary Figure 3 — Baseline and follow-up cytokine levels of patients who had myalgia vs. people without any symptoms at the follow-up. [file Image_3.JPEG]

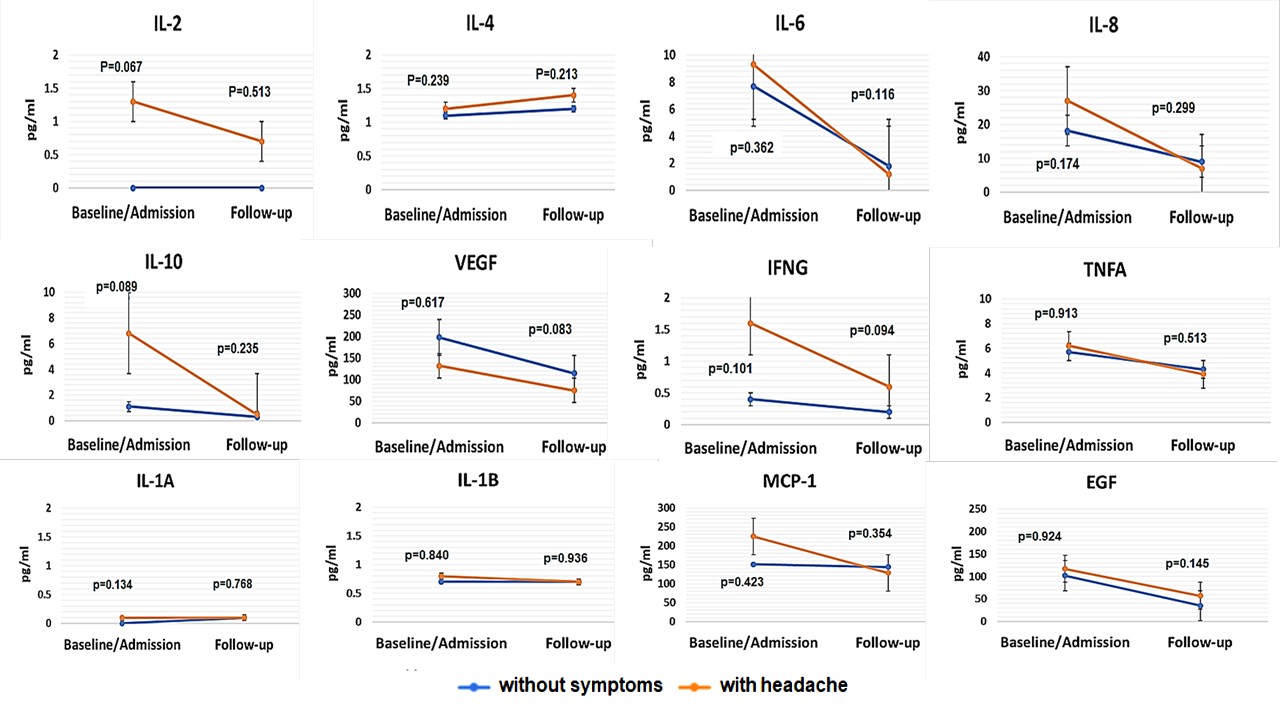

Supplement: Supplementary Figure 4 — Baseline and follow-up cytokine levels of patients who had headache vs. people without any symptoms at the follow-up. [file Image_4.JPEG]

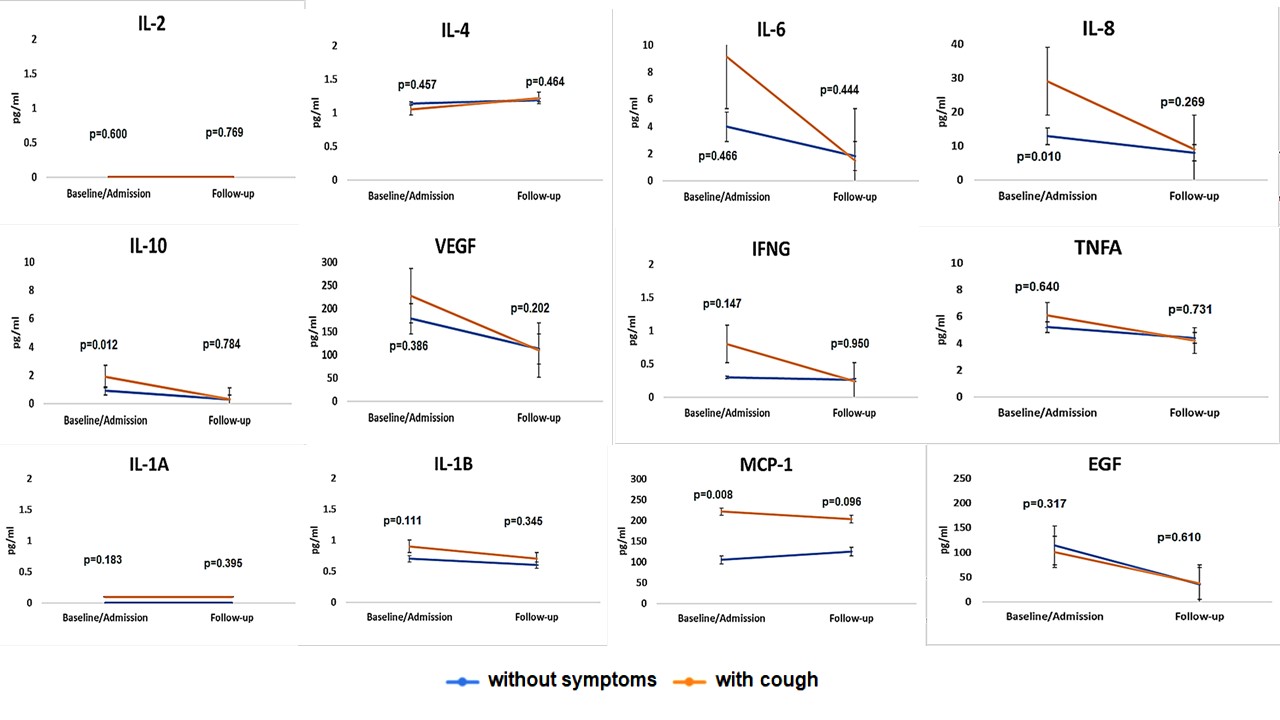

Supplement: Supplementary Figure 5 — Baseline and follow-up cytokine levels of patients who had cough vs. people without any symptoms at the follow-up. [file Image_5.JPEG]

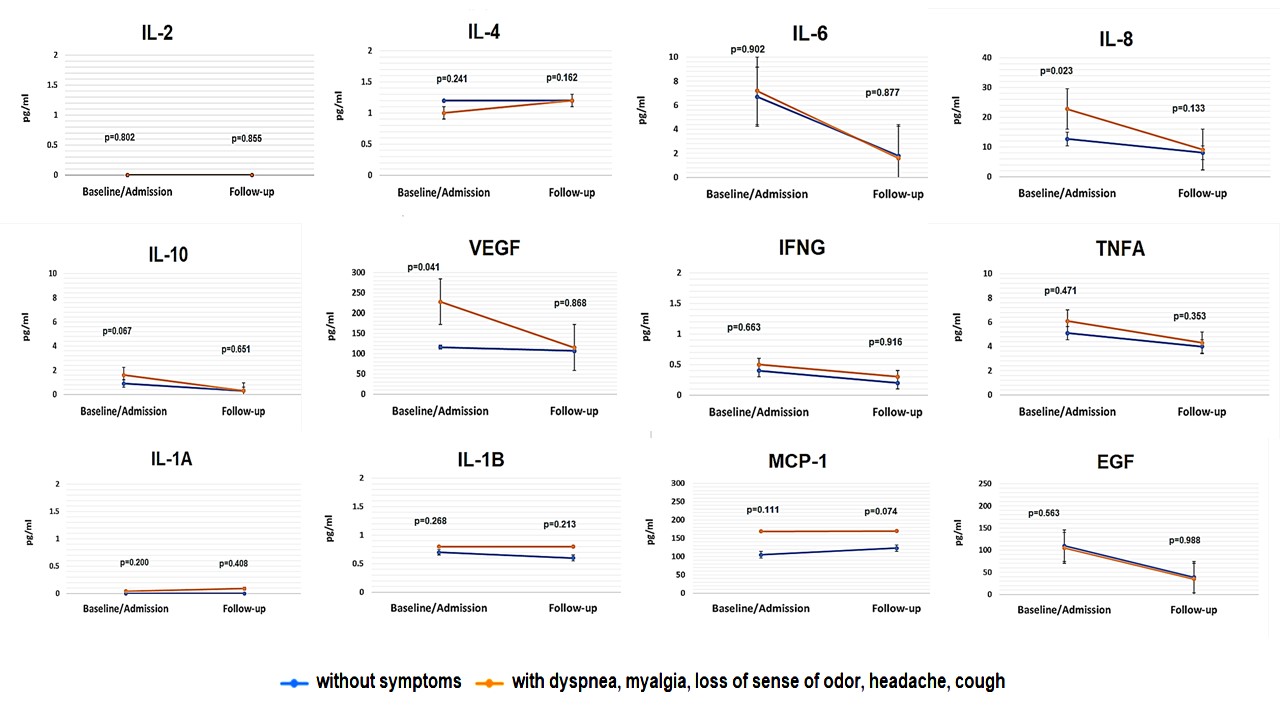

Supplement: Supplementary Figure 6 — Baseline and follow-up cytokine levels of patients who had either dyspnea, loss of sense of odor, myalgia, headache, or cough vs. people without any symptoms at the follow-up. [file Image_6.JPEG]
